# The role and attitude of senior leaders in promoting group-based community physical activity: a qualitative study

**DOI:** 10.1186/s12877-020-01795-2

**Published:** 2020-10-02

**Authors:** Hiroko Komatsu, Kaori Yagasaki, Yuko Oguma, Yoshinobu Saito, Yasuhiro Komatsu

**Affiliations:** 1 Japanese Red Cross Kyushu International College of Nursing, 1-1 Asty, Munakata-City, Fukuoka-Prefecture 811-4157 Japan; 2 Faculty of Nursing and Medical Care, Keio University, 35 Shinanomachi, Shinjuku-ku, Tokyo, 160-8582 Japan; 3 Sports Medicine Research Center & Graduate School of Health Management, Keio University, 4-1-1 Hiyoshi, Kohoku-ku, Yokohama, Kanagawa 223-8521 Japan; 4 Center for Innovation Policy, Kanagawa University of Human Services, Research Gate Building TONOMACHI 2-A, 3-25-10 Tonomachi, Kawasaki-ku, Kawasaki-shi, Kanagawa 210-0821 Japan; 5 Graduate School of Medicine, Gunma University, 3-39-22 Showa-machi, Maebashi, Gunma 371-8511 Japan

**Keywords:** Older adults, Physical activity, Senior leader, Social participant, Healthy longevity

## Abstract

**Background:**

In the context of worldwide public health, it is very important to promote physical activity among the older people. This study explored the roles and attitudes of senior leaders in promoting group-based exercise in their local communities, specifically to determine the level and extent to which to elderly participation was encouraged.

**Methods:**

This study conducted semi-structured face-to-face in-depth interviews and employed a subsequent thematic analysis. Participants included 10 club leaders and five sub-leaders who were working at senior clubs in Fujisawa-city, Kanagawa, Japan, from July to September 2018.

**Results:**

Four themes emerged from the interview responses, including “unwavering attitude/conviction in relation to the vision,” “leaders must set an example,” “a search for balance in delegating responsibilities to members,” and “creating and fostering culture and environment of mutual help.” Further, each participant outlined several aims, including “achieving healthy longevity for the entire local community,” “having older people promote healthy activities among the older people,” and “creating a pro-health town.”

**Conclusions:**

Findings indicate that policymakers, public health workers, and healthcare providers should recognize the pivotal roles that senior group leaders play in promoting healthy activities for the older people. These efforts should be strongly considered when developing policies and strategies designed to promote overall healthy longevity from a general community perspective.

## Background

As the world population continues to age [1], it becomes increasingly important to maintain physical, cognitive, and social competencies among older adults. Indeed, the older people demographic has become a critical target for public health interventions [2]. In this regard, regular physical activity (PA) provides a range of health benefits for older adults. For instance, it is known to reduce the risk of developing heart disease, stroke, type 2 diabetes, and some cancers [3]. Further, accumulated evidence specifically shows the health benefits of regular PA for older adults [4], and many guidelines now promote PA for seniors [5, 6]. However, PA participation remains low among older adults in many nations, including the United States, Canada, and the United Kingdom [7]. Studies have also shown that community-wide PA interventions have stronger effects on behavioral change compared to individually tailored interventions [8, 9]. Our previous work on community-wide physical activity showed that more adults in the intervention group were aware of PA guidelines than those in the control group at baseline [10]. Further, effective community-wide programs can encourage regular PA among all community members while increasing levels of social engagement [11].

On the other hand, Baker et al. found no consistent evidence to support the effectiveness of multi-component community-wide interventions designed to increase populational levels of PA. Further, a number of related problems were identified [12]. Still, research has found that some strategies aimed at facilitating PA may improve the reach and effectiveness of interventions at the community level. For instance, this includes interventions that involve existing community groups and/or community spokespersons [13]. Meanwhile, other research has emphasized that older people can maintain health by achieving social capital through social engagement via activities such as group exercise [14]. Furthermore, our previous work have already found that older adults can achieve balanced health in the physical, mental, and social domains through regular group exercises as part of a community-wide PA intervention, which also contributed to community expansion through social connectedness and mutual support [15]. Leaders within the senior-citizen community thus play essential roles in generating social capital. However, the essential elements and styles needed for such leadership have not yet been elucidated.

This study explored the roles and attitudes of senior leaders in promoting group-based community PA at senior clubs in Fujisawa-city, Kanagawa, Japan, specifically to examine whether those efforts facilitated social participation among the older people.

## Methods

### Design

This was a qualitative study that collected data through individual interviews. Then, a thematic analysis was employed to identify and report related themes [16]. Taken together, these processes elucidated the experiences of leaders and assistant leaders working at senior clubs participating in the Fujisawa + 10 exercise program. The study design was approved by the Institutional Ethics Review Board of the Graduate school of Health Management, Keio University (2017–12).

### Communitywide intervention for increasing PA: Fujisawa + 10

Japan’s aging national society is now progressing to a degree that is seen nowhere else in the world. The Japanese government has thus officially recommended that local governments empower their communities and develop social capital to promote community health. From this perspective, Fujisawa City aims to achieve a more elder-friendly social organization by promoting the achievement of the goal “Creation of a society where people and city administration collaborate and support each other,” “Respect for individual’s dignity and identity,” and “People can keep living independently in their familiar communities,” based on the basic principle of “Creation of a city where people support each other, being healthy in mind and body.” [17]. More specifically, Fujisawa City is heavily promoting increased PA at the populational level by encouraging such activities among citizens. Here, the key message is found through the city’s “Plus Ten” slogan (i.e., citizens should remain active for 10 more minutes than now). In collaboration with Fujisawa City, this study’s researchers developed the “Fujisawa +10 exercise program” as part of a communitywide intervention campaign [10]. Exercise intensity is low enough to accommodate an intervention that introduces proper PA habits to older adults in the community [10]. The exercises were introduced to this demographic through voluntary group-based programs demonstrated at the city center and local community parks. Attendees were taught how to exercise and then given CDs, DVDs, and printed manuals to help them engage in PA without the need for an instructor. Senior leaders working at local senior clubs played the central roles throughout this process.

### Setting and participants

Study participants were sampled purposively among senior club leaders and assistant leaders who were working with older adults who participated in regular group-based exercises as part of a community-wide PA intervention. One researcher (either YO or YS) briefly explained the study purpose to each participant. If they agreed to be interviewed, arrangements were then made to meet at a specific date, time, and location. First, each participant was introduced to one of the investigators (either HK or KY), who provided further details. Investigators and participants then met in a private room at a public meeting hall. Participants were also given written and oral explanations of the study objectives, methods, and voluntary nature of their participation.

All participants (*n* = 15) provided their oral and written informed consent prior to participation.

### Data collection

A semi-structured interview guide was developed to gain insight into how participants perceived the implementation of community-wide group-based exercises (supplementary file 1). Opportunities for additional discussion were also provided.

Interview items and questions included the following:

(i) Please tell us about the roles you play as a leader in order to continue your activities, including group-based community exercise.
(ii) What rules and/or guidelines should be followed? What other things require care and attention when implementing group exercises and other activities?
(iii) As a leader, do you feel that you carry a heavy burden? On the other hand, is there anything that provides you with a sense of doing something worthwhile, such as having a mission or being valued?
(iv) What essential elements are required of a leader (for instance, competence) when initiating and maintaining group-based community activities? Alternatively, what sorts of issues are important for you and your group?

After obtaining their informed consent, a total of 15 participants (including leaders and assistant leaders) were interviewed in a private room at a public meeting hall. The specific interview dates chosen by each respective participant. One of the investigators (either KY or HK) conducted the interviews between July and September 2018. One postgraduate student was also present at each interview for observation purposes. Each participant was interviewed once, with all interviews audiotaped and then transcribed verbatim. After interviewing 13 participants, with the last two interviews presenting no new theme, KY and HK determined that data saturation was reached and thus terminated interviews.

### Data analysis

All transcripts were reviewed and thematically analyzed. KY reviewed each transcript several times and anonymized any personally identifiable information. Following previously established thematic analysis phases [16], KY then 1) independently and continuously reviewed the transcripts, 2) extracted and initially coded relevant data from the entire data set, 3) identified candidate themes by repeatedly comparing and integrating individual codes and reporting models in the data, 4) identified appropriate themes by reviewing the candidate themes, 5) determined subthemes, and 6) selected quotations for use in the results section.

To ensure study trustworthiness, we also adopted the following procedures: KY analyzed the data and noted personal perceptions and instances of subjectivity by marking them with parentheses. HK then reviewed the initially coded data and tentative subthemes. Next, all themes and subthemes were discussed; final themes were established by both KY and HK. After completing all interviews, the research team held two peer debriefings to discuss the study themes and interpretations while soliciting the opinions of public health professionals HK and YO.

The COREQ checklist [18] was used to ensure the quality of reporting in this study (Additional file 1).

## Results

This study interviewed 15 total participants (10 leaders and five assistant leaders) who were working at senior clubs in Fujisawa-city, Kanagawa, Japan. The clubs held regular group-based exercise sessions under the Fujisawa + 10 program, along with other activities. Participants included 12 males and three females with a mean age of 77.3 (range of 69–85), while mean interview duration was 39.6 min (range of 26–90 min). A subsequent analysis identified the following themes:

(i) Unwavering attitude/conviction in relation to their vision.
(ii) Leaders must set an example.
(iii) A search for balance in delegating responsibilities to members.
(iv) Creating and fostering a culture and environment of mutual help.

Each participant had several aims, including achieving healthy longevity for their entire local communities, promoting healthy activities among the older people by soliciting help from within the older people community, and creating a pro-health town.

### Consistent attitude/conviction in relation to their vision

Many senior club leaders held the same vision; that is, under the slogan “Our community as the first place of healthy longevity in Japan/promoting healthy longevity.” These participants exhibited strong goal-oriented leadership to achieve these goals through consistent beliefs and attitudes. The interviews revealed that it was not easy to run the senior clubs. In this regard, participants confronted a wide range of issues, such as maintaining a sufficient number of club members, continuing activities, and ensuring succession between generations. However, all participants were convinced that consistent, unwavering attitudes and convictions toward the goal of achieving better health and wellbeing among local citizens was a driving force for sustaining senior club activities. Further, they hoped to achieve health and peace among all community members, not solely among their club members. One said the following:

*“To grow together - grow together in the community - we need to think of ourselves as sharing a common destiny. Every day, I pray for healthy longevity without accident for everyone in our community. I really think that way. Of course, this is for the development and growth of our club. I think that way every morning” (M).*

Participants recognized that group-based physical exercise promoted not only physical health, but also psychological health. As such, these types of activities were regularly included:

*“Even though I run the activities every week, when I leave home in the morning, my feeling is like a vision. The significance seems to me to relate to not only the physical exercises, but also emotional health” (J).*

On the other hand, participants who were responsible for running senior clubs often confronted major challenges when recruiting and retaining club members. For instance, Leader K said that this was the most persistent difficulty related to their club responsibilities. Several participants also recognized the importance of consistent and unwavering attitudes/convictions when facing different and sometimes contrary opinions expressed by local citizens. Leader B said:

*“One point to emphasize is to never give up. What is giving up? For example, suppose I have an idea, but when I talk about it, people don’t approve. Will I give up? No, I won’t. I withhold my proposal, and after a while, I can make a proposal, can’t I? That approach seems necessary to me” (B).*

Some interviewees said that club leaders working at large organizations reported their activities on website homepages. In this regard, public relations were also deemed important. Leader O said:

*“Third parties express various opinions [text omitted], and when hearing those the leader must be completely unwavering. Instead of changing their attitudes, leaders must believe in their visions, and convey the visions, basic principles, and activities to others” (O).*

### Leaders must set an example

In terms of leadership style, many participants did not explicitly provide one-to-one exercise instruction to their members, but instead demonstrated movements in front of groups of older people. These deliberate presentations served as models that participants could follow, thus offering guidance and leadership. Leader H said:

*“It is one example of leadership (it is the leadership), isn’t it? The only way is to do things yourself first. It depends on the situation, though. If you just explain things verbally, people won’t move. To some extent, at least, you often have to go out there and do things yourself, without speaking” (H).*

Similarly, Leader J said:

*“As to how to lead and involve people, there really is no alternative to showing your feelings and your personality, is there? (nothing works better than demonstrating your vision, commitment, and openness, does it?)” (J).*

Participants recognized that a given leader’s attitude and behavior (e.g., not enforcing member participation, but quietly providing a model to follow) could influence whether they gained trust. Leaders J and I said:

*“The leader is no good if he or she is not humble” (J).*

*“That’s the role of a leader, isn’t it? When he or she says something, the members trust it. Trust is what matters” (I).*

The leader’s own practices and efforts were also essential when demonstrating a model for members to follow. Participants said that leaders must properly study and practice exercises in their own homes. That is, they must maintain strong efforts even while in private. Leaders H and A said:

*“I definitely need to become more knowledgeable and study more” (H).*

*“I was enthusiastic about learning about these. I found out what Plus Ten was from watching videos” (A).*

Some of the assistant leaders recognized that the leader’s presence and attitude motivated them to participate in or cooperate with a club. Leader C said:

*“I think that Leader B, the Chairperson, perhaps had a very hard time when he started to manage the club. They presented lots of ideas, and so on, and that sort of thing. We all felt that since the Chairperson was so enthusiastic, we had to help them by doing whatever we could” (C).*

Participants also recognized that leaders could not manage club activities by themselves. For that reason, it was essential to have assistants or subleaders (titles varied between clubs). Because these individuals served as advisers, leaders derived strength from them and developed close ties, similar to those seen in large organizations. Leaders I and J said:

*“While the leader is important, definitely important, the presence of a subleader who acts as a reliable second-in-command - giving candid advice and following the leader - is important as well. Without such a subleader, the leader is unable to satisfactorily achieve their goals. [text omitted] Therefore, the presence of a subleader who fully supports the leader - enables the leader to fully demonstrate their leadership - makes an excellent organization” (I).*

*“I think that what is needed is not a single strong, charismatic leader, but one who serves and is supported by those around them, with mutual help” (J).*

It was important for leaders of all titles to share a vision about running the club while working together closely. Leader O said:

*“Leadership members - the leader, subleader, and such - need to communicate and resolve any conflicts, to share a common vision. They should avoid conflicts or expressing different opinions” (O).*

### A search for balance in delegating rights, roles, and responsibilities to club members

Declining membership numbers and the closures of other senior clubs also required participants to change their leadership styles. That is, they attempted to balance the degree of delegation. At times, this involved the avoidance of shifting too many responsibilities so that members would not feel overwhelmed. At other times, they delegated some responsibilities to members, thus preventing attitudes that were too dependent or passive. They also indicated that roles should be delegated based on a careful assessment of this balance while considering people’s feelings. In this context, participants recognized that they should delegate roles and responsibilities as appropriate; that is, based on an understanding of individual attributes, abilities, and deficiencies. Leader G said:

*“Nowadays, more people decline to join the senior clubs because they don’t want to take on responsibility. Several senior clubs have closed in many areas. People don’t want to take the leading roles. They feel the burden is too great and don’t want to take on any more duties” (G).*

On the other hand, they also realized that club activity was highly supported through autonomy and the delegation of certain responsibilities and roles: Leader J said:

*“It’s obviously good to see people share responsibilities” … “We all know each other, and their skills and their weakness” (J).*

Participants recognized that the deliberate delegation of responsibilities and roles created independent attitudes among members. Leader M said:

*“By doing this on an individual basis, so that each person is given the appropriate roles - how shall I put it? - this means that people who were previously rather passive became stronger and more assertive” (M).*

On the other hand, leaders from several clubs reported difficulty in maintaining activities. One leader recognized that club decline was partly caused by tedious routines. Leaders M and G said:

*“I can do this enthusiastically for about one year, but will find it difficult to continue thereafter” (M).*

*“I always wondered whether there was something else to add, but I definitely understand that [text omitted] just doing the exercises alone leads to the activities becoming tediously routine and uncreative” (G).*

### Creating and fostering a culture and environment of mutual support

Participants recognized that older people should not solely be supported by younger people. Rather, they also need support from peers and must be responsible for developing activities while maintaining and promoting health. For this reason, participants recruited local citizens who shared their ideas. This vision was not limited to mutual support, but also extended to more dynamic activities and programs through which older people could cooperate with younger people to build healthy communities. This provided a change in their involvement and subsequent enjoyment. Leaders L and B said:

*“Above all, everyone getting together. When I first took the role of the leader, I told all the senior club members that we were like brothers and sisters. People become isolated if they live alone and talk to no one, don’t they? If you find someone like that, it’s important to talk to them, isn’t it?” (L).*

*“Now, how can we older people support other older people who need it? Of course, support targets not just food, but also how to spend everyday life. That includes checking whether they are alive or dead, doesn’t it? We must need to consider those kind of things. Actually, we ourselves enter the stage when we need support from others. But we need to recognize that” (B).*

Leader H learned about research showing that social networking promoted healthy longevity. He therefore valued socialization among the older people, who could learn new things and enjoy themselves. Leader H said:

*“We often hear that communities and human connections are getting weaker. I also felt that way, and thought that another important aspect of the senior club was enabling older people to collaborate in studying and enjoying things together” (H).*

Furthermore, they engaged in cooperative “Engawa” activities with other senior clubs. Engawa activity is an informal meeting or activity conducted at community public spaces that people can freely access and use. Engawa is an element of the traditional Japanese house that provides a platform for social networking. Many local governments, including the Fujisawa government, have built spaces that citizens can freely use to promote social networking. Many local government including Fujisawa city build space where citizens can freely use to promote social networking. This required members of all age groups and residential areas to meet and talk. Leaders I and B said:

*“What I value most now is the opportunity to meet with young people, something like a business networking meeting, where people with different backgrounds can meet. Different kinds of people who could not have the chance to get together at traditional senior club meetings or early morning radio physical exercise programs” (I).*

*“As a part of a residents’ association, we started the ‘people’s circle’. The term may not be correct, but it is an Engawa activity, which Fujisawa city is promoting. It connects the older people and children. We signed up for that, and have been conducting it since last year” (B).*

Another participant discussed a pioneering project to introduce information and communication technology into the local community. This participant was concerned with population declines in residential areas and worried that older residents were physically deteriorating. They established challenges for the future based on changing societal circumstances rather than simply maintaining current activities Leader B said:

*“Information and communication technology is not the area I have mainly focused on [i.e., introducing information and communication technology]. However, technology must be used. Must. Basically, this idea has been always on my mind [text omitted]. You know, when Fujisawa City introduced robot taxis for the first time in Japan, members of this club supported that” (B).*

## Discussion

Many societies are continuing to age throughout the world. This makes civic participation and engagement an area of increasing importance, with many countries reexamining their approaches to dealing with aging [19, 20]. While civic engagement is widely accepted and discussed as part of a conceptual framework for increasing social capital, little research has attempted to identify the phenomena related to the roles of social capital, social cohesion, and social engagement [21]. As such, this study focused on group-based communitywide exercise programs in order to examine the pivotal roles that senior group leaders played in health promotion and whether their efforts enhanced social participation among the older people.

Senior club leaders typically establish a vision. For instance, this may include “becoming the number one place in Japan for healthy longevity,” or simply “promoting healthy longevity.” In this regard, leadership ability is demonstrated through consistent attitudes/convictions toward achieving these visions. Leaders must have firm convictions about the need to achieve healthy longevity in their communities rather than simply aiming to maintain their own personal health. Publicly shared visions thus become basic principles for leadership actions. Further, such leadership is voluntary. This is important because individuals are more motivated to volunteer based on expressions of humanitarian value and thus more likely to engage [22]. When leaders proclaim their visions, as mentioned above, they become core group values. These values are then reinforced by older adults who volunteer, thus generating human, social, and cultural capital [23].

Participants said that consistent and unwavering beliefs and attitudes helped to establish group cohesion; as such, the refusal to give up was pivotal for sustaining club activities. Because group activities were run on a volunteer basis, leaders felt that the abandonment of a leadership role could lead to group decline and club closure. Further, participants were from the “Baby Boom” generation (i.e., born after World War II), and had thus been responsible for postwar rebuilding efforts, thus establishing the most rapid period of economic growth in Japanese history. We can therefore assume that these individuals shared the common principles of perseverance, endurance, and unwavering belief. In turn, this helped them overcome hardships.

In terms of leadership style, most participants did not provide one-on-one exercise instruction to members, but instead demonstrated movements in front of whole classes. In this way, they humbly served as models and guides. Leaders are thus positive role models who increase participation by instilling effectiveness and confidence in members while providing guidance for appropriate behavior [24, 25]. At the same time, participants sometimes found it difficult to motivate participants to exercise. In this regard, the importance of assistants and subleaders became clear; these individuals shared responsibilities and discussed issues with the club leaders. Leaders and their assistants therefore established strong bonds by sharing responsibilities, which then increased group effectiveness through the implementation of mutually promoted and coordinated activities. Indeed, shared responsibility and group commitment can be associated with success [26].

Participants also learned from cases in which nearby senior clubs failed to retain members and finally suspended club activities. In this regard, they delegated certain roles and responsibilities to club members, thus providing opportunities for independent and voluntary engagement without creating heavy burdens. Participants recognized that they could specifically delegate roles based on the personal traits, abilities, and deficiencies of members.

Further, great efforts were made to build group cohesion by maintaining appropriate connections with members. This also required consideration of their individual personal traits, abilities, and deficiencies. In other words, cohesion was maximized when members recognized shared characteristics. This was achieved by focusing on veterans and delivering interventions at community sites, where members were likely to share geographical commonalities [26]. Furthermore, the leaders made efforts to develop programs that can continuously capture the interests of participants. For example, in addition to physical activity (Plus 10), they introduced recreational activities such as table tennis and traditional Japanese card games. These activities not only entertained the participants but also provided challenges that allowed participants to experience personal growth. The evolving, dynamic nature of this program is key for its success.

Leaders built and maintained cohesion by expecting participants to engage in independent activities (i.e., avoiding passive attitudes), thereby achieving social engagement through delegated responsibilities and maintaining balance. This empowered individuals to become group members. For older persons, empowerment can stem from mutual support systems in which group members promote efficacy, mastery, and control despite the presence of ageist structural barriers [27]. Leaders ensured that group efficacy increased by understanding the abilities and deficiencies of club members while establishing appropriate levels of support and balance. In turn, this increased the likelihood of group empowerment and social action, both of which carry over to individual members by providing a sense of ownership in overall group success [28].

Participants knew that great efforts were required to maintain and promote health among club members. This required not only ensuring that the older people received support from younger people, but that they also received mutual support from their peers. Participants also established active groups comprised of members with diverse background and ages by meeting with local citizens and striving to both learn from and teach them while achieving mutual understanding. Participants thus saw group evolution through expressions of ideas, opinions, emotions, and feelings, which were evident in their interactions, trust levels, and shared confidence [29]. In this regard, leaders gained a sense of efficacy and empowerment by positively shaping the societal view of older adults. At the same time, leaders felt that their groups created “experiential confidence,” or a common experience of success. This continued to serve as a platform for confidence when setting and achieving goals [28]. Leader B said:

*“To fully consider the problem of populational decline in the local community and the deterioration of physical function among the older people, we introduced information and communication technology into local communities. We want to take on the new challenge of adapting to changing societal needs rather than simply maintaining the status quo” (B).*

Various leadership styles are adopted among entities such as businesses, governments, healthcare organizations, and communities. This study found that servant leadership was predominant among senior leaders of group-based communitywide physical exercise programs. Servant leadership has been defined as “a philosophy and set of practices that enriches the lives of individuals, builds better organizations and ultimately creates a more just and caring world” [30–33]. Other researchers have identified the seven servant leadership behaviors of conceptualizing, emotional healing, putting followers first, helping followers grow and succeed, behaving ethically, empowering, and creating value for the community [34]. Here, conceptualization refers to an individual’s ability to act as a visionary for their organization, thus providing a clear sense of its goals and directions [33]. This study’s participants exhibited these behaviors, thereby demonstrating servant leadership when organizing physical exercise activities in their communities.

### Limitations

This study had several limitations. First, the study sample was small. Further, selection bias may have occurred due to purposeful sampling, while the themes produced may have been influenced by the imbalanced proportions of male and female participants (12 and 3, respectively). In our study, we did not collect detailed background social history of participants, including past leadership experience and duration, their past job, and whether they had any relevant work experiences in the physical activity area. This information may aid in the interpretation of the leaders’ experiences. Finally, this study was conducted in Japan, a country with large aging population. Thus, the attitude and responses of older individuals may differ from those of older individuals in other countries. This may limit generalizability of our findings.

## Conclusions

This study focused on group-based community-wide exercise programs to examine the pivotal roles that senior leaders played in health promotion and whether older participation was thus enhanced. Participants had several aims, including the achievement of healthy longevity for their entire communities, helping older citizens promote activities among their peers, and creating a pro-health town. Our findings suggest that policymakers, public health workers, and healthcare providers would benefit from recognizing these pivotal roles in promoting healthy activities by adopting related strategies when developing programs designed to increase healthy community longevity.

## Supplementary information

**Additional file 1.** Consolidated criteria for reporting qualitative studies (COREQ): 32-item checklist.

**Additional file 2: Supplementary file 1.** Semi-structured interview guide

## Data Availability

The datasets used and/or analysed during the current study available from the corresponding author on reasonable request.

## Abbreviations

PA: Physical activity
COREQ: Consolidated criteria for reporting qualitative research.
