# Supplementary material for: The role and attitude of senior leaders in promoting group-based community physical activity: a qualitative study
Source: BMC Geriatr. 2020 Oct 2;20:380. doi: 10.1186/s12877-020-01795-2 (PMC7532647; doi:10.1186/s12877-020-01795-2)
Supplement: Supplementary file 1 — Additional file 1. Consolidated criteria for reporting qualitative studies (COREQ): 32-item checklist. [file 12877_2020_1795_MOESM1_ESM.docx]

**Additional file 1: Consolidated criteria for reporting qualitative studies (COREQ): 32-item checklist**

Title：The role and attitude of senior leaders in promoting group-based community physical activity: A qualitative study

**Authors:** Hiroko Komatsu, RN, PhD., Kaori Yagasaki, RN, PhD., Yuko Oguma, MD, PhD., Yoshinobu Saito, PhD, Yasuhiro Komatsu, MD, PhD.

| **No. Item** | **Guide questions/description** | **Response** |
| --- | --- | --- |
| **Domain 1: Research team and reflexivity** | | |
| *Personal Characteristics* | | |
| 1. Interviewer/facilitator | Which author/s conducted  the interview or focus group? | Hiroko Komatsu (HK)  Kaori Yagasaki (KY) |
| 2. Credentials | What were the researcher’s  credentials? E.g. PhD, MD | KY and HK: RN, PhD  YO: MD, PhD  YA: PhD  YK, MD, PhD |
| 3. Occupation | What was their occupation at the time of the study? | HK: Professor and President  KY: Professor  YO: Associate Professor  YS: Project researcher  YK: Professor |
| 4. Gender | Was the researcher male or  female? | Three females and two males |
| 5. Experience and training | What experience or training  did the researcher have? | HK and KY have done previous several qualitative research projects in oncology. |
| *Relationship with participants* | | |
| 6. Relationship established | Was a relationship  established prior to study  commencement? | HK, KY, and YK had not relationships with participants. In other study, YO and YS had relationships with all participants. |
| 7. Participant knowledge of the interviewer | What did the participants  know about the  researcher? e.g. personal  goals, reasons for doing the  research | Participants knew about the interviewer’s names and facility, and the objectives of research and interviews. |
| 8.Interviewer characteristics | What characteristics were  reported about the  interviewer/facilitator? e.g.  bias, assumptions, reasons  and interests in the  research topic | See manuscript in the method section. |

| **Domain 2: study design** | | |
| --- | --- | --- |
| *Theoretical framework* | | |
| 9. Methodological orientation and Theory | What methodological  orientation was stated to  underpin the study? e.g.  grounded theory, discourse  analysis, ethnography,  phenomenology, content  analysis | Thematic analysis.  See manuscript in the method section. |
| *Participant selection* | | |
| 10. Sampling | How were participants  selected? e.g. purposive,  convenience, consecutive,  snowball | Purposive sampling.  See manuscript in the method section. |
| 11. Method of approach | How were participants  approached? e.g. face-to face, telephone, mail, email | Face-to-face.  See manuscript in the method section. |
| 12. Sample size | How many participants were in the study? | 15 participants.  See manuscript in the results section. |
| 13. Non-participation | How many people refused  to participate or dropped out? Reasons? | No one has dropped out. |
| *Setting* | | |
| 14. Setting of data collection | Where was the data collected? e.g. home, clinic,  workplace | We were interviewed in a private room at a public meeting hall.  See manuscript in the method section. |
| 15.　Presence of  nonparticipants | Was anyone else present  besides the participants  and researchers? | None |
| 16. Description of sample | What are the important  characteristics of the sample? e.g. demographic data, date | See manuscript in the method section. |
| *Data collection* | | |
| 17. Interview guide | Were questions, prompts,  guides provided by the  authors? Was it pilot  tested? | Interview guide was not pilot tested.  See manuscript in the method section. |
| 18. Repeat interviews | Were repeat interview  carried out? If yes, how  many? | No |
| 19. Audio/visual recording | Did the research use audio  or visual recording to  collect the data? | Yes. The interviews were audio recorded.  See manuscript in the method section. |
| 20. Field notes | Were field notes made  during and/or after the  interview or focus group? | No |
| 21. Duration | What was the duration of  the interviews or focus  group? | See manuscript in the results section. |
| 22. Data saturation | Was data saturation  discussed? | Yes. KY and HK discussed the data saturation. |
| 23. Transcripts returned | Were transcripts returned  to participants for comment  and/or correction? | No. |

| **Domain 3: analysis and findings** | | |
| --- | --- | --- |
| *Data analysis* | | |
| 24. Number of data coders | How many data coders coded the data? | Two, KY and HK. |
| 25. Description of the coding tree | Did authors provide a  description of the coding tree? | No |
| 26. Derivation of themes | Were themes identified in  advance or derived from the data? | Yes. We identified key themes emergent from the data.  See manuscript in the method section. |
| 27. Software | What software, if applicable, was used to manage the data? | No |
| 28. Participant checking | Did participants provide  feedback on the findings? | No. |
| *Reporting* | | |
| 29. Quotations presented | Were participant quotations  presented to illustrate the  themes/findings? Was each  quotation identified?  e.g. participant number | Yes.  See manuscript in the results. |
| 30. Data and findings  consistent | Was there consistency  between the data presented and the findings? | Yes. We reviewed the data with all the themes. All the final themes were established at peer debriefing among the investigators. |
| 31. Clarity of major themes | Were major themes clearly  presented in the findings? | Yes. See manuscript in the results. |
| 32. Clarity of minor themes | Is there a description of diverse cases or discussion of minor themes? | No. |

Developed from: Tong A, Sainsbury P, Craig J. Consolidated criteria for reporting qualitative research (COREQ): a 32-item checklist for interviews and focus groups. *International Journal for Quality in Health Care*. 2007. Volume 19, Number 6: pp. 349 – 357
