# Supplementary material for: The role and attitude of senior leaders in promoting group-based community physical activity: a qualitative study
Source: BMC Geriatr. 2020 Oct 2;20:380. doi: 10.1186/s12877-020-01795-2 (PMC7532647; doi:10.1186/s12877-020-01795-2)
Supplement: Supplementary file 2 — Additional file 2: Supplementary file 1. Semi-structured interview guide [file 12877_2020_1795_MOESM2_ESM.docx]

Supplementary file 1.

Semi-structured interview guide

| 1. Please tell me about your role as a leader in maintaining community group exercise and other activities.  2. What are the important requirements for maintaining group activities? Which of them do you think are the most important?  3. Please tell me about the burdens, accomplishments, sense of mission, or values that you experienced during this process.  4. Please tell me about the difficulties and challenges you faced while maintaining and managing the group exercise program.  5. What do you think are the essential elements and competencies a leader should possess to continue community group activities?  6. Please tell me about the challenges faced by you and your group. |
| --- |
